# An Integrated Model of the Transcriptome of HER2-Positive Breast Cancer

**DOI:** 10.1371/journal.pone.0079298

**Published:** 2013-11-01

**Authors:** Krishna R. Kalari, Brian M. Necela, Xiaojia Tang, Kevin J. Thompson, Melissa Lau, Jeanette E. Eckel-Passow, Jennifer M. Kachergus, S. Keith Anderson, Zhifu Sun, Saurabh Baheti, Jennifer M. Carr, Tiffany R. Baker, Poulami Barman, Derek C. Radisky, Richard W. Joseph, Sarah A. McLaughlin, High-seng Chai, Stephan Camille, David Rossell, Yan W. Asmann, E. Aubrey Thompson, Edith A. Perez

**Affiliations:** 1 Department of Cancer Biology, Mayo Clinic, Jacksonville, Florida, United States of America; 2 Department of Biomedical Statistics and Informatics, Mayo Clinic, Rochester, Minnesota, United States of America; 3 Department of Internal Medicine, Division of Hematology and Oncology, Mayo Clinic, Jacksonville, Florida, United States of America; 4 Department of Surgery, Mayo Clinic, Jacksonville, Florida, United States of America; 5 Institute for Research in Biomedicine, Barcelona, Espirito Santo, Spain; King's College London, United Kingdom

## Abstract

Our goal in these analyses was to use genomic features from a test set of primary breast tumors to build an integrated transcriptome landscape model that makes relevant hypothetical predictions about the biological and/or clinical behavior of HER2-positive breast cancer. We interrogated RNA-Seq data from benign breast lesions, ER+, triple negative, and HER2-positive tumors to identify 685 differentially expressed genes, 102 alternatively spliced genes, and 303 genes that expressed single nucleotide sequence variants (eSNVs) that were associated with the HER2-positive tumors in our survey panel. These features were integrated into a transcriptome landscape model that identified 12 highly interconnected genomic modules, each of which represents a cellular processes pathway that appears to define the genomic architecture of the HER2-positive tumors in our test set. The generality of the model was confirmed by the observation that several key pathways were enriched in HER2-positive TCGA breast tumors. The ability of this model to make relevant predictions about the biology of breast cancer cells was established by the observation that integrin signaling was linked to lapatinib sensitivity in vitro and strongly associated with risk of relapse in the NCCTG N9831 adjuvant trastuzumab clinical trial dataset. Additional modules from the HER2 transcriptome model, including ubiquitin-mediated proteolysis, TGF-beta signaling, RHO-family GTPase signaling, and M-phase progression, were linked to response to lapatinib and paclitaxel in vitro and/or risk of relapse in the N9831 dataset. These data indicate that an integrated transcriptome landscape model derived from a test set of HER2-positive breast tumors has potential for predicting outcome and for identifying novel potential therapeutic strategies for this breast cancer subtype.

## Introduction

Approximately 15% of all invasive breast tumors, at presentation, overexpress the EGFR family member HER2 [1–3]. Clinically, this subset of breast tumors is defined by high level expression of HER2 on the plasma membrane of >10% of the cells within a tumor, assessed by immunohistochemistry, and/or by amplification of the *ERBB2* gene, as evidenced by fluorescent in situ hybridization. High level HER2 expression is associated with decreased overall survival [3,4]. Several large clinical trials have shown that patients with such HER2-positive tumors benefit from HER2-targeted therapies. The initial targeted trials were done with the humanized HER2 monoclonal antibody trastuzumab (Herceptin®), first in the metastatic and then in the adjuvant setting [5–12]. Such targeted therapy in the adjuvant setting has resulted in a dramatic increase in survival of patients with HER2 breast cancer, as first firmly established by clinical trials such as NCCTG N9831 and NSABP B31 [13], which have helped define the standard of care for such patients. Additional trials have been carried out (or are in progress) using other HER2 monoclonal antibodies (pertuzumab, trastuzumab-emtansine) as well as small molecule receptor tyrosine kinase inhibitors (lapatinib) that target HER2 signaling activity.

 Although tremendous strides have been made in management of patients with HER2-positive tumors, a number of important questions remain to be answered about this clinical subtype of breast cancer. Although there is abundant evidence that HER2-positive tumors manifest distinct patterns of gene expression, alternative splicing, and somatic mutation [14] [15] [16] [17] [18], the basic biology of this tumor subtype is not well understood. We do not fully understand the processes that are activated downstream of HER2 gene amplification and overexpression. It is likely that these HER2-associated processes affect the manner in which tumors respond to HER2-targeted therapy and/or to conventional chemotherapy in combination with HER2-targeted therapy; so identification of key processes that are critical to the establishment and maintenance of HER2-positive tumors may inform novel therapeutic strategies to overcome primary or acquired resistance to HER2-targeted therapies or lead to the development of alternative therapeutic strategies that are less expensive than trastuzumab, which is in many cases beyond the means of patients in underdeveloped countries. 

We reasoned that the key to understanding the clinical behavior of HER2-positive tumors lies within networks of interacting genes that affect the activity of biological processes that are essential to establishment and maintenance of the HER2-transformed phenotype. Thus, our analyses were motivated by the central concept that the clinical/biological properties of the tumors will be defined not by individual genes but by the processes that are controlled by multiple interactive genomic features. To evaluate this hypothesis we interrogated total (polyA+) RNA sequence (RNA-Seq) data from a test set that consisted of benign breast tissue samples plus ER+, triple negative, and HER2-positive primary invasive breast tumors. We identified genes that are differentially expressed, transcripts that are alternatively spliced, and genes that express novel, non-synonymous single nucleotide polymorphisms. We integrated these genomic features to model the interactions between such HER2-associated genomic features in our test set. This model was comprised of 12 highly interconnected genomic modules, each of which corresponds to a distinct functional process. The generality of this model was tested by interrogation of gene expression data from TCGA breast cancer samples, and the ability of the model to make relevant biological predictions was evaluated using data from the Cell Line Encyclopedia as well as clinical outcomes data from an adjuvant trastuzumab trial (N9831). Our results reveal that novel functions related to integrin signaling, ubiquitin-mediated proteolysis, M-phase progression, RHO-family GTPase signaling, and TGF-beta signaling are linked to drug response and to clinical outcome in HER2-positive tumors. 

## Materials and Methods

### Samples

Polyladenylylated RNA was isolated from epithelial cells derived from excisional biopsies of 8 breast lesions that were subsequently determined to be free of DCIS or invasive cancer. These samples will hereinafter be called “benign”. RNA was also prepared from 8 fresh-frozen tissues of ER+ (estrogen receptor positive), 8 HER2+ (HER2-enriched), and 8 triple negative (TN – estrogen receptor negative, HER2 negative, and progesterone receptor negative) tumors (all infiltrating ductal carcinoma). Clinical/pathological characteristics of these tumors are given in Table S1. RNA quality of the samples was determined using Agilent Bioanalyzer and all samples had a RIN value > 7.9. cDNA libraries were prepared and sequenced (2 X 50nt paired ends) by the Mayo Clinic Genome Facility using the Illumina Genome Analyzer II (GAIIx). The RNA sequence data used in this manuscript have been previously used to call fusion transcripts from tumor samples [19]. All patients gave full written consent for these studies. Protocols for tissue collection (MC09-001909) and sequence analysis (MC09-000035) were approved by the Mayo Clinic Institutional Review Board. Data used in this study are deposited in the Gene Expression Omnibus web site at GSE45419.

### Databases and software used for analyses

FASTQ files from RNA sequencing were aligned to the human genome build NCBI 37.1 (GRCh37), which corresponds to human genome assembly hg19 in UCSC [20]. BWA [21] and TopHat [22] alignment software were used to align paired-end RNA-Seq reads. The 1000 Genomes Project (1000g 2011May) and 5400 exome sequencing data were obtained for filtering single nucleotide variants from UCSC genome browser. Germ-line single nucleotide variants were filtered using dbSNP version 135 (http://www.ncbi.nlm.nih.gov/SNP/). Functional annotation of SNVs was performed using the ANNOVAR software [23]. A variety of statistical packages from R programming language were used for computing and generating some plots, including JMP 9.0 (http://www.jmp.com/) and Partek6.6 (http://www.partek.com/). A Microsoft SQL server database was used to store and query nucleotide positions to call SNVs. PERL and R programming language was used to write subroutines for data analysis. 

### Alignment

Paired-end RNA-Seq reads were aligned to both genome and exon-exon boundary databases using BWA to obtain gene counts. BWA is a fast and accurate short read aligner [21]. Reads with more than two mismatches in first 32 bases in each alignment and reads that mapped to multiple genomic locations (alignment score less than 3) were discarded. In order to make confident single nucleotide variant calls from RNA-Seq data, we have used two aligners (BWA and Bowtie). Bowtie is an ultrafast and memory-efficient short read aligner that does not allow gaps. TopHat is a fast splice junction mapping software that uses Bowtie alignment to align RNA-Seq reads [22,24]. Binary Alignment/Map (BAM) format files from BWA and TopHat were obtained for each sample. Gene counts were derived from both TopHat and BWA aligners using BED tools from UCSC to count reads corresponding to a known gene. The correlation between the gene counts was high from TopHat and BWA aligners (r =0.95, range 0.94 to 0.97). Hence, we have used BWA gene counts for gene expression analysis. We used TopHat BAM files for both alternative splicing analysis and SNV analysis. Consensus SNV calls were obtained for samples if they were present from both BWA and TopHat pileup files, as described below. 

### Gene Expression analysis

**Figure 1:**
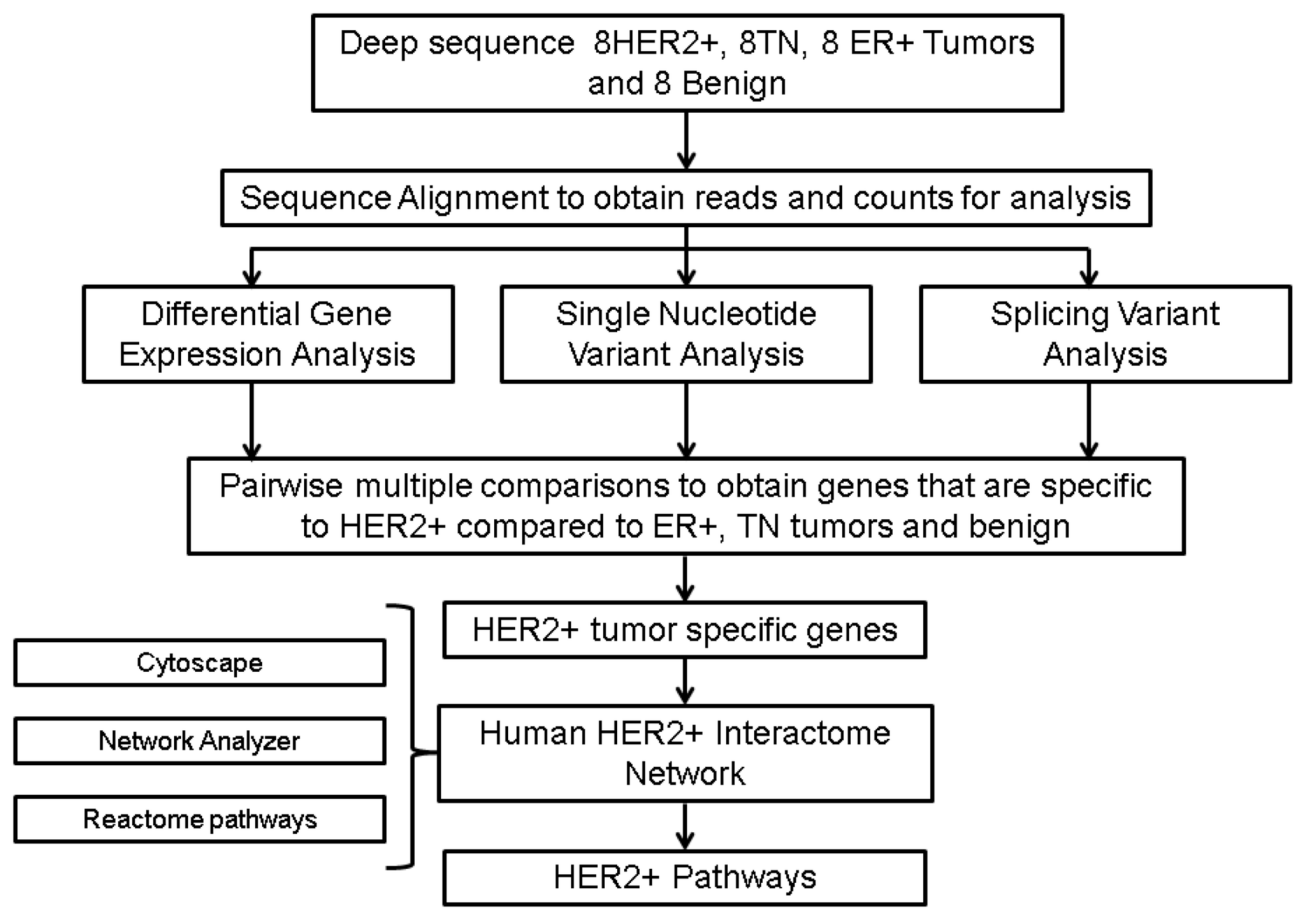
Methods workflow for HER2 transcriptomic network. High level analytical approach to build HER2-positive transcriptomic landscape from paired-end RNA-Seq data analysis of breast tumor subtypes.

Figure 1 summarizes the approach to identification of HER2-associated genomic features in our test set of tumor samples. Gene counts for samples were summarized and annotated using our in-house scripts developed using BamToBed utility and UCSC refFlat annotations for further analysis. The read counts for genes were obtained for downstream differential gene expression analysis. There were a total of 22,323 genes with gene count data for normalization. Individual gene count data were normalized using mode normalization method as previously described [25]. Genes that had a median read count >2^4^ (16 reads) in at least one of the four groups were used for gene expression analysis. After removing the genes with low expression, there were 16,195 genes considered for differential gene expression analysis. The Dunnett-Tukey-Kramer (DTK) package in R programming language was used for pairwise multiple comparison tests for unequal variance and unequal sample sizes [26]. Genes for which the HER2 tumor group had a mean log (read+1) significantly different from the means of the other tumor and normal groups were obtained. A HER2-positive gene list (p<0.05) was obtained after filtering multiple comparison values from HER2+, ER+, TN, and benign groups. 

### Alternative splicing analysis

BAM files after alignment were loaded into Bioconductor. CASPER, implemented as an R package (https://sites.google.com/site/rosselldavid/software), was used to quantify known splicing variants. Maximum likelihood estimates of the relative abundances of known transcripts were obtained via the Expectation Maximization (EM) algorithm using CASPER as previously described [25]. Genes that were expressed (gene read count > 16) and had more than one alternatively spliced transcript were used to determine the ratios of transcript expression values in each sample. Group comparisons of transcripts were performed using the Dunnett-Tukey-Kramer package, as described above for gene expression analysis. Transcripts for which the HER2-positive tumor group had a mean ratio significantly different from ER+, TN, and benign cohorts were identified. 

### Expressed single nucleotide variant (eSNV) analysis

**Figure 2:**
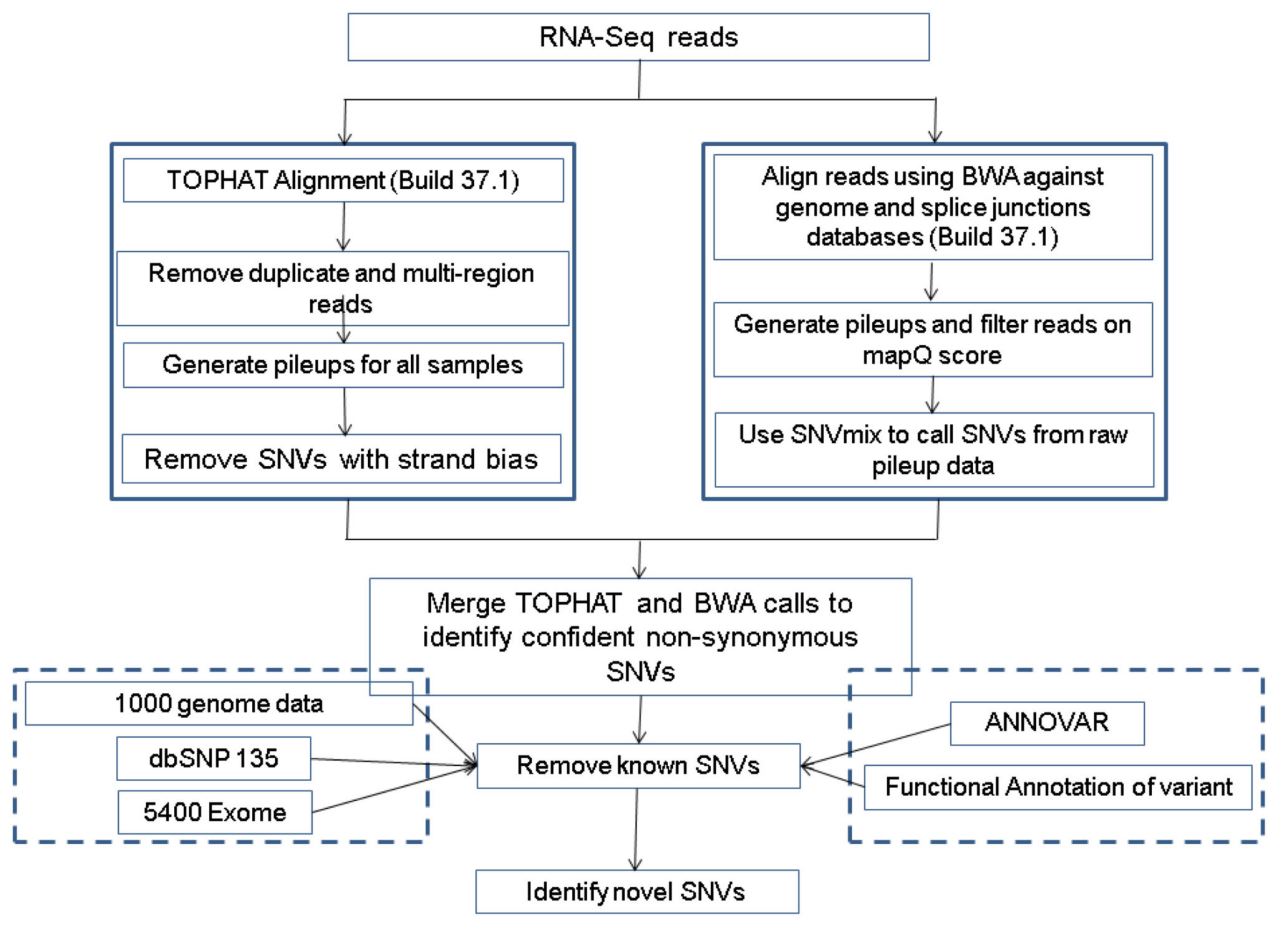
The eSNV-Detect workflow. Flow chart of the eSNV-Detect – a method to identify expressed SNVs from paired-end RNA-Sequencing data.

RNA-Seq data were used to detect expressed nucleotide sequence variants (eSNVs), including somatic mutations. In general, genotype calling algorithms for SNVs were designed for germline DNA and not for RNA. Hence, the false detection rates to call an SNV are high in RNA-Seq data from tumors. We have developed an eSNV-Detect (expressed single nucleotide variant detection) workflow with parallel alignment strategies (Bowtie and BWA). The advantage of using two aligners in eSNV-Detect is that we take complementary strengths of two aligners and reduce the weakness of these tools. Figure 2 shows the flowchart of the workflow for eSNV-Detect. We have used eSNV-Detect, to call novel SNVs in tumors using a combination of computational tools and methods. We merged both junction and genomic alignment reads from BWA and generated one pileup file for each sample as previously described [25]. The pileup file generated from each BWA BAM file was used to predict SNVs using SNVMix software [27]. Reads with low quality (mapQ score < 20) were filtered by SNVMix. We obtained the reference allele, number of reads supporting reference call, alternate allele, and number of reads supporting alternate allele, for every transcribed position for which evidence of an alternate allele was present. We also filtered reads for read depth <4 and ratio of alternate allele <0.1 (ratio of alternate allele = number of reads with alternate allele/total read depth). TopHat (Bowtie) analysis was used to eliminate duplicate reads and multi-region hit reads from the BAM file. We generated a pileup file from the filtered BAM file. For each nucleotide position, we obtained the reads supporting A+, A-, C+, C-, G+, G-, T+ and T-, where + and - indicate the polarity of the read strand. For a position with alternate allele evidence from BWA, we made sure that the variant called is not due to strand bias by making use of TopHat data. For example if the variant allele is G, we examined the sequences of both G+ and G- data to ascertain that the variant allele was present on both the strands and that the ratio of alternate allele count on positive and negative strand was >0.1. After removing the SNVs that exhibited strand bias, we obtained the eSNV candidates that were confidently called by two approaches. Consensus SNVs were obtained and filtered based on annotation as shown in our eSNV-Detect workflow (Figure 2). Only the eSNVs that were present from two approaches and were non-synonymous were carried further for analysis. The final eSNVs obtained from our method were filtered for germline or known SNVs present in dbSNP 135, 1000 genome, or 5400 exome datasets. After a series of annotation filters as shown in Figure 2 were applied, we identified novel eSNVs for each sample. All the eSNVs identified were stored in a MySQl database along with the tumor type for a sample. The database was queried to obtain eSNVs that were detected only in HER2-positive. Finally, HER2-positive eSNVs were evaluated for 3’ or 5’ end bias using the data from pileup files; alternate allele reads uniquely detected at either 5’ or 3’ ends of the reads were eliminated using VCF tools.

### Network analysis

Genomic features (differential expression, alternative splicing, and eSNVs) that were differentially detected in the HER2-positive tumors included in the test set were compiled for network analysis using Cytoscape [28]. Network analysis was performed in Cytoscape using the Reactome FI feature [29] and network analyzer [30] plug-ins to construct an interactome model. Linker genes (not present in our list but known to interact with our integrated gene list based on literature evidence) were excluded from the HER2 network to emphasize the degree of direct connectivity between and among our HER2-positive genomic features. Comprehensive network parameters such as number of edges for each node, distribution of degree counts and neighborhood connectivity were obtained using network analyzer in Cytoscape. We also performed clustering analysis in Cytoscape to identify modules or clusters in the HER2 network. The Pathway Enrichment analysis tool in Cytoscape was used to identify pathways enriched in each module of the HER2 network. 

### Cell Line Encyclopedia data analysis

Gene expression and pharmacodynamic data for paclitaxel and lapatinib were obtained for cancer cell lines from the cancer cell line encyclopedia (CCLE) data sets [31]. Of note is that we did not include trastuzumab data, as Cell Line Encyclopedia data for trastuzumab are not available. There were 58 breast cancer cell lines in the encyclopedia database, of which 20 have paclitaxel response data (EC50) and 18 have lapatinib response data (EC50). Spearman log rank correlation analysis was used to identify HER2 network genes that correlated with EC50 values for these two drugs.

### Analysis of association between genes in the HER2 network and clinical outcome following HER2-targeted therapy

DASL expression array data were obtained from the NCCTG N9831 adjuvant trastuzumab clinical trial for 1282 patients for whom we have clinical response data [12]. All patients in the N9831 study were randomized to receive the anthracycline doxorubicin plus cyclophosphamide followed by paclitaxel alone (Arm A), paclitaxel followed by trastuzumab (Arm B), and concurrent paclitaxel plus trastuzumab (Arm C) [13]. Gene Set Analysis [32] was carried out using data from Arm C and genes from the HER2 transcriptome model to determine if any of the modules within this network are associated with risk of relapse, defined by univariable Cox Hazard ratio analysis of individual genes from the DASL array dataset versus time to event (distant relapse) as a continuous variable [33].

### The Cancer Genome Atlas (TCGA) validation of candidate pathways

Gene count data from TCGA breast tumor samples (RNA-Seq) were downloaded from the TCGA data portal. PAM50 definitions of intrinsic subtype were used to assort samples into HER2-enriched (n=66), basal-like (n=140), and luminal (n=604) cohorts. DTK analysis was carried out using TCGA gene counts for differentially expressed genes from our initial analysis of 32 tumors. Fisher’s exact test was used to determine if genes associated with relevant pathways were significantly enriched in HER2 samples from TCGA. Gene copy number and mutational data from TCGA breast tumors were extracted from Cbioportal (www.cbioportal.org).

### RT-PCR and Sanger sequencing validation

Genomic DNA from tumors was extracted from the BCT40 HER2 tumor and tumor adjacent normal tissue, and 83 eSNVs were validated using Sanger sequencing. Primer pairs for the eSNVs were designed using Primer3 version 4.0 software, and were used to amplify all variants by PCR. PCR products were purified from unincorporated nucleotides using a Millipore PCR purification plate. A total volume of 10μl, containing 80ng of the purified PCR product and 1.6pM of one of the primers (forward or reverse), was used for sequencing. Electropherograms were analyzed with SeqScape v2.5 (ABI, Applied Biosystems, Foster City, CA, USA). Quantitative real time PCR (qPCR) was used to verify two alternative splice isoforms of MPG transcripts (MPG-002, MPG-201) in tumor and benign samples. Equal aliquots of total RNA from samples were converted to cDNA with the High-Capacity cDNA Archive kit (Applied Biosystems), and qPCR reactions were performed in triplicate with 10ng of cDNA and the TaqMan® Universal PCR master mix (Applied Biosystems). Custom primer/probe sets were purchased from Applied Biosystems: NM_001015052.1 forward primer GGTCCGAGTCCCACGAA, reverse primer CTGGTCGCTGCTTCTTTTGC, reporter CCCAGTTTTGCCGACGGATG; NM_001015054.1 forward primer GTGCCCAGTTGCTCTTCAAG, reverse primer CTGGTCGCTGCTTCTTTTGC, reporter CCGTCGGCAAAACTGAAAA. All amplification data were collected with an Applied Biosystems Prism 7900 sequence detector and analyzed with Sequence Detection System software (Applied Biosystems). Data were normalized to POLR2A, and mRNA abundance was calculated using the ΔΔCT method. 

## Results

**Figure 3:**
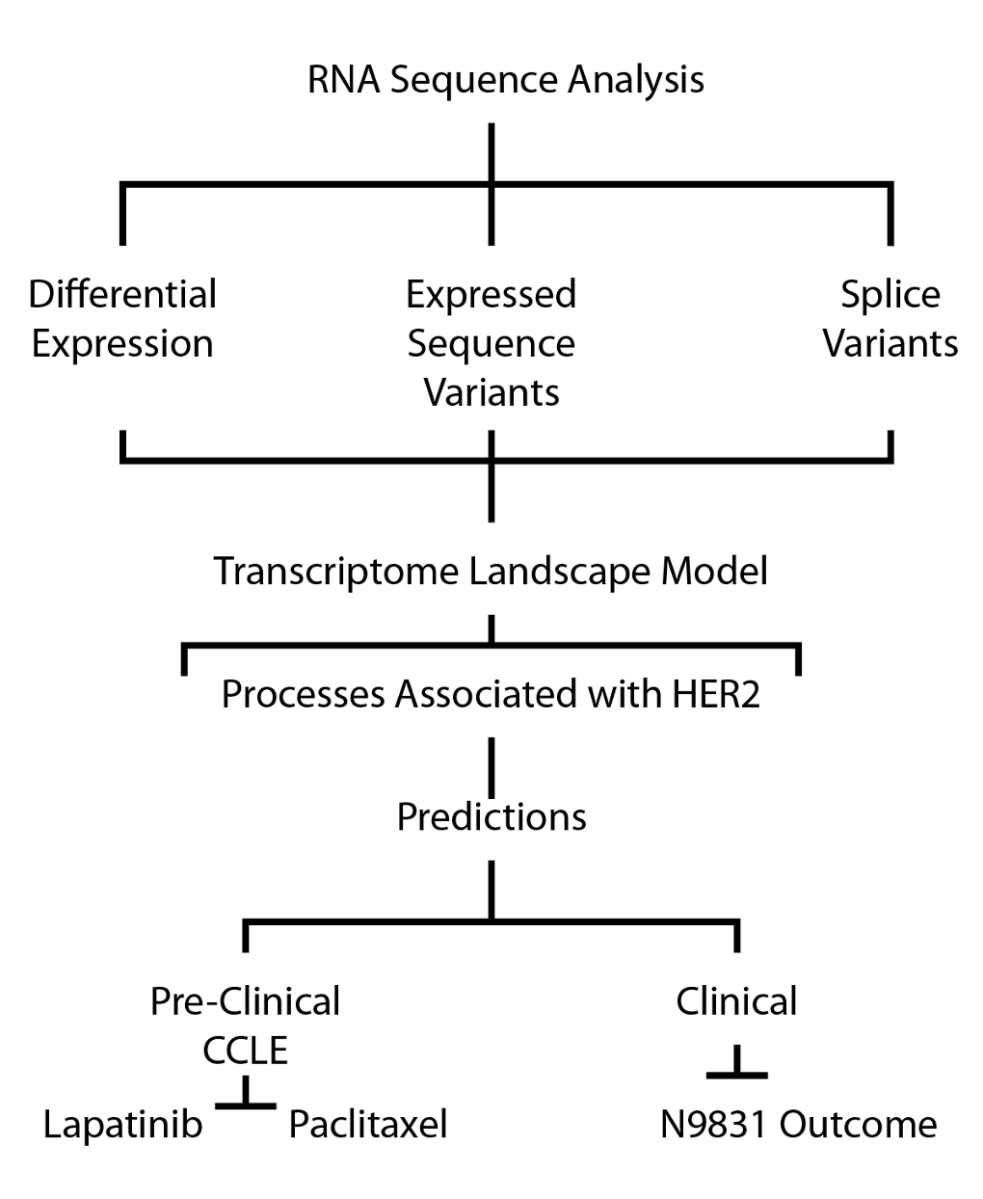
Schematic of analytical approach. Computational approach to identify and characterize genomic features associated with HER2-positive tumors.

The analytical plan, outlined in Figure 3, began with paired end (2x50nt) RNA sequence (RNA-Seq) analysis of polyA+ RNA from a survey panel of samples that consisted of 8 benign breast tumor samples plus 8 each ER+, HER2-positive, and triple negative (TN) primary invasive breast tumors. Sequence alignment data are summarized in Table S2; but, briefly, percent aligned reads ranged from 73-86%, with GeneCount-ReadStart aligned ranging from 22M to 68M read pairs per sample. (Note that for paired end sequencing, total aligned 50nt tags is equal to twice the number of read pairs.) Mode normalization of gene counts was carried out as described previously [25]. As shown in Figure S1, density plots indicated that genes with <16 total aligned tags were near or below the limits of detection; and these were eliminated from the analyses. Transcripts with >16 tags aligned ranged from 14-17K per sample, with the exception of one outlier, a TN tumor with only 11,230 transcripts (ID - BR73 in Table S2). Principle components analysis indicated that this tumor did not cluster with the TN tumors, or any of the other tumor cohorts (not shown); this sample was excluded from all subsequent analyses. 

 As indicated in Figure 3, the summarized RNA-Seq data were used (as described below) to identify genes that were differentially expressed or alternatively spliced in the HER2-positive tumors in our test set, as well as genes that contained expressed single nucleotide variants that were detected only in this HER2-positive tumor cohort. Genes associated with such features were used to build an integrated transcriptome landscape map that identified a number of biological processes that appear to be predominant in the HER2-positive tumors included in this analysis. 

### Identification of genomic features that define the HER2-positive tumors in our test set

#### Differential expression

**Figure 4:**
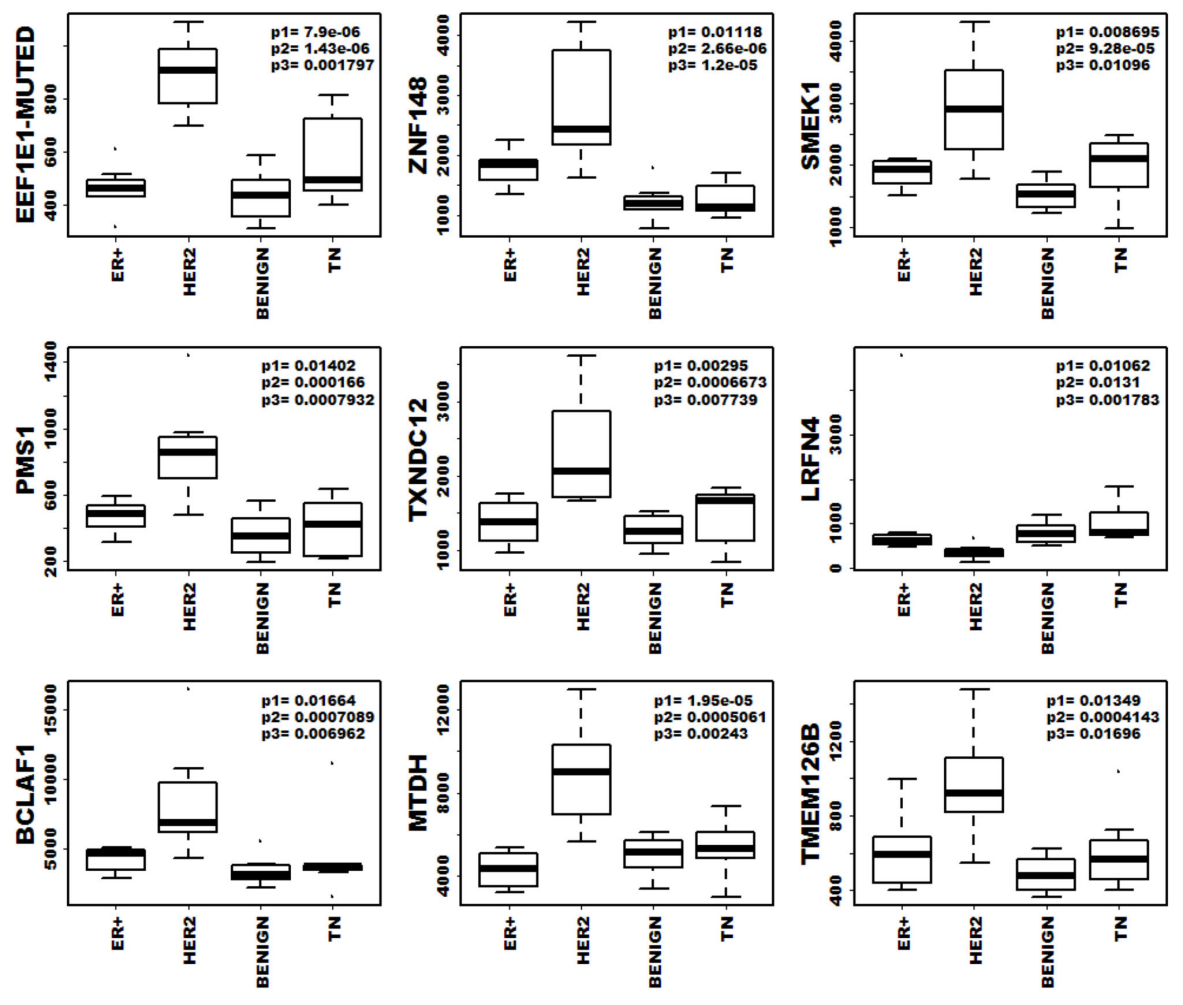
Top 9 differentially expressed genes. Box plot representation of nine differential expressed genes that are specific to the HER2-positive tumors compared to other tumors in our test set, shown in log_2_ scale.

**Figure 5:**
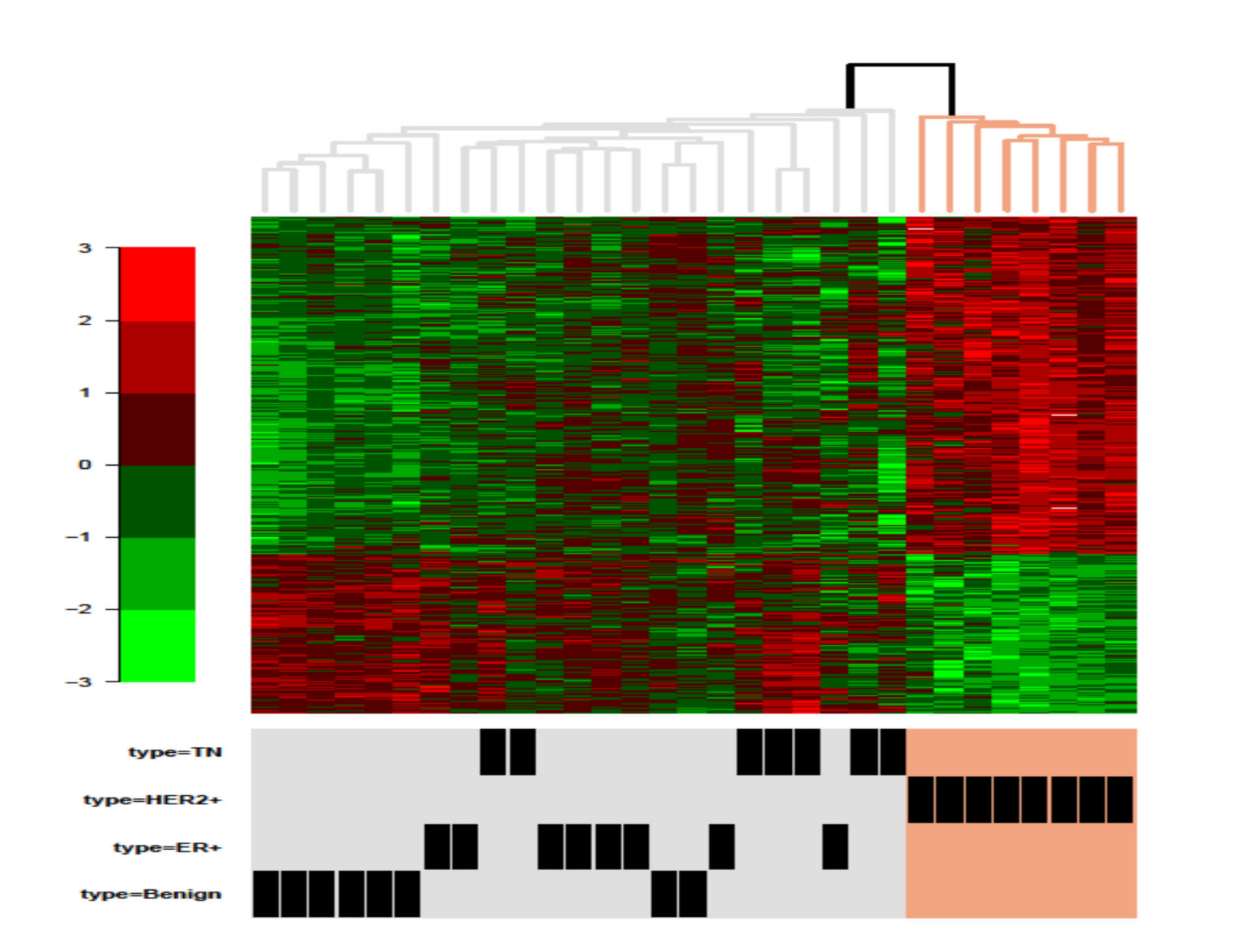
Clustering of genes uniquely expressed in HER2 tumors. Hierarchical clustering of 685 significantly differentially expressed genes in HER2-positive tumors compared to other tumor subtypes in our test set of tumors.

Mean gene expression was compared between the tumor types using the Dunnett-Tukey-Kramar procedure, which controls type1 error for all pair-wise comparisons between the groups. We used mode normalized, log_2_ transformed mRNA abundance of individual genes in the four sample cohorts (HER2, ER+, TN, benign). We identified 685 genes that were differentially expressed in these HER2-positive tumors at p<0.05 compared to each of the other breast tumor subtypes in the test set. Nine representative genes are shown in Figure 4, and the remainder in Table S3. Hierarchical clustering (Figure 5) graphically illustrates the distribution of all 685 genes in the tumors analyzed in this cohort. 

#### Alternative splicing

**Figure 6:**
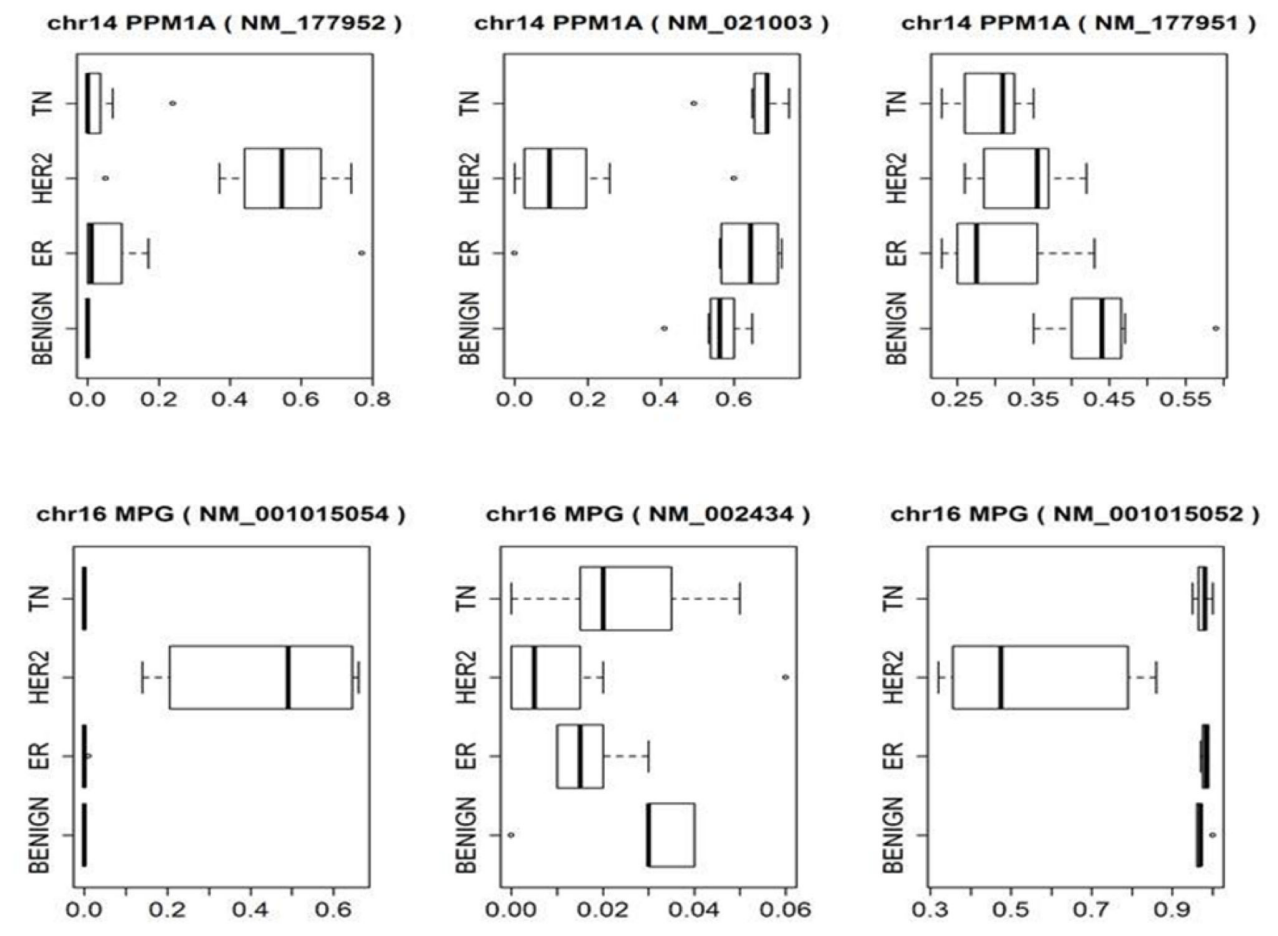
CASPER plots for 2 genes. Visualization of output values from CASPER splicing analysis method for PPM1A and MPG genes. Data indicates that PPM1A and MPG transcripts are uniquely and alternately spliced in HER2-positive tumors compared to other groups.

The activity of a given gene is influenced not only by changes in the abundance of the gene product (mRNA), but also by changes in the nucleotide sequence of the transcript. Alternative splicing can give rise to protein products with different functions, such that differential expression of splice variants represents one potential mechanism to affect changes in gene activity. The CASPER package was used to quantify known RefSeq splice variants in the four sample cohorts. The relative abundance of each alternatively spliced isoform was calculated as counts assigned to an individual isoform/total counts for all transcript isoforms for each gene. Splice variants in the four sample cohorts were identified by Dunnett-Tukey-Kramar analysis of the relative (fractional) abundance of the isoforms. Although CASPER interrogates only known splice variants, we have previously validated the utility of this tool for identifying alternatively spliced genes in lung cancer samples [25]. Representative data from two genes (PPM1A and MPG) that are alternatively spliced in a pattern that was observed in the HER2-positive tumors are shown in Figure 6. For example, PPM1A splice variant NM_177952 is relatively abundant in our test set of HER2-positive tumors whereas NM_021003 is relatively abundant in the benign, ER+, and TN tumors included in this group of samples. A total of 199 transcripts, corresponding to 102 genes, were alternatively spliced in a manner that was uniquely associated with our HER2-positive tumors (Table S4). 

#### Expressed single nucleotide sequence variants

 In addition to alternative splicing, it is clear that genetic (mutational) events play a significant role in the generation of gene products with altered functions in tumor cells. Identification of expressed single nucleotide sequence variants (eSNVs) from RNA-Seq data has been challenging due to the dynamic range of RNA abundance, which imposes significant limits upon the confidence with which one can identify such features in transcripts that are expressed at low abundance. Additional issues related to sequence strand, exon junction, and 5’ end bias are also common sources of false positive detection, as discussed in the Materials and Methods section. As described on Figure 2, we have developed a novel analytical workflow that addresses and eliminates most of these issues. 

 Using the filters described in Materials and Methods, we identified 318 high confidence novel exonic non-synonymous eSNVs that were expressed at >3 counts of the alternate allele in one or more of our HER2-positive tumors but not in benign, ER+, or TN samples or in any of the SNP databases (e.g. dbSNP135, 1000 genome, or 5400 exome). These candidate novel eSNVs are listed in Table S5. The HER2-associated eSNVs correspond to 303 genes, listed in Table S6. The range of high confidence novel eSNVs per HER2-positive tumor was 22-83, median 35 (Table S7). Median eSNVs that were unique to our ER+ and TN tumor samples were 34 and 57 respectively, with no statistically significant differences in eSNV/tumor for any of the subtypes.

 We used an earlier version of our eSNV workflow to call *KRAS* mutations in lung adenocarcinoma samples with 100% accuracy [25]. We selected one HER2-positive tumor to validate the current version of this analytical tool in breast cancer. Tumor BCT40 was selected because it had the largest number of candidate eSNVs among the HER2-positive cohort, with 83 high-confidence candidates nominated (Table S8). Total read depth for these 83 candidates ranged from 6 to 327 reads, and alternate allele reads ranges from 4 to 93 reads. PCR amplification followed by Sanger sequence analysis of genomic DNA from tumor and tumor-adjacent normal tissue confirmed 79 of these candidate eSNVs, indicating that our false detection rate for this analysis is in the range of about 5%. The three candidates that did not validate had minimal read depth (4 alternate allele reads); however, 6/9 candidates with alternate read depth = 4 validated, and all candidates with 5 or more alternate reads were validated. We conclude that filtering for 4 or more alternate allele reads gives us an acceptable false detection rate on the order of 5%. 

 Since germ line (blood) DNA was not available for these samples, we used tumor-adjacent normal tissue for identification of potential somatic mutations. Among the 79 validated eSNVs, 51 were unique to the tumor genomic DNA, whereas 28 were detected in both tumor and tumor-adjacent normal tissue. Although all of the eSNVs were filtered to remove known polymorphisms (present in dbSNP135, 1000 genome, and 5400 exome datasets), our data suggest that at most 1/3 of our candidate eSNVs may be rare germ line polymorphisms. This conclusion must be advanced with caution, since tumor-adjacent tissue may be contaminated with tumor cells. Be that as it may be, we conservatively predict that at least two-thirds of our candidate eSNVs are bona fide somatic mutations. 

**Figure 7:**
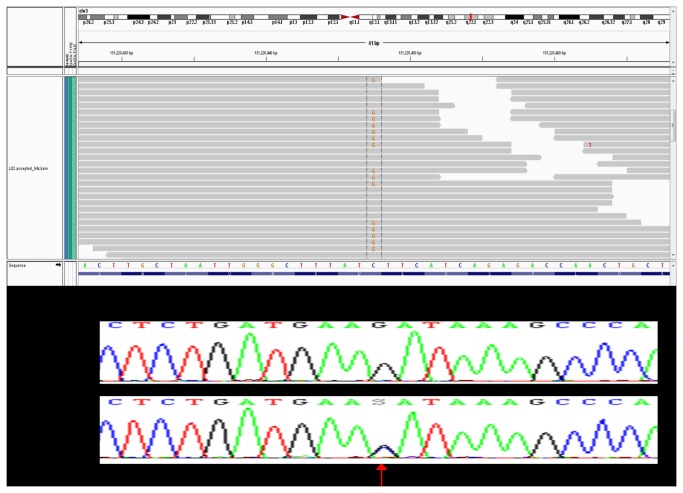
Visualization of single nucleotide variant validation. Sanger sequence validation of highly expressed novel somatic SNVs for *MRPL3* variant in the BCT40 HER2 tumor sample. RNA-Seq sequence reads shown above Sanger sequencing tracing, with mutation shown by an arrow.

**Figure 8:**
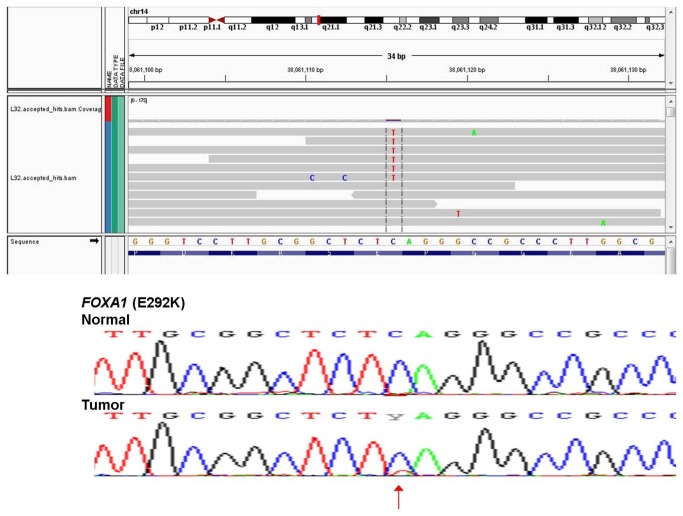
Representation of single nucleotide variant validation. Sanger sequence validation of low expressed novel somatic SNVs for *FOXA1* in the BCT40 HER2 tumor sample. RNA-Seq sequence reads shown over Sanger sequence trace with mutation indicated by an arrow.

 Sanger sequence chromatograms for two validated somatic mutations are shown in Figures 7 and 8 to illustrate the range of allelic frequencies that we commonly observed. *MRPL3* was nominated in tumor BCT40 as a C to G variant on chromosome 3 at coordinates 131220447 (chromosome 3: 131220447) with 53 reads supporting the reference allele (C) and 79 reads supporting the alternate allele (G). Sanger sequence analysis (Figure 7) confirmed this as a heterozygous D69H (chromosome 3: 131220447) somatic mutation. In contrast, the gene encoding transcription factor FOXA1 was called as a C to T variant (chromosome 14:38061115) with 4 reference allele reads and 6 alternate allele reads. Although this variant was confirmed as a somatic mutation by Sanger sequence analysis (Figure 8), the variant allele appears to be present in less than half of the alleles in tumor genomic DNA. This observation informs our strong supposition that RNA-Seq is a very sensitive tool for detecting eSNVs that are expressed in a subset of tumor cells. 

Nine of the HER2-restricted eSNVs were predicted to be nonsense mutations (“stopgain” in Table S5, column J “Exonic Function”). This group included *TP53*, *AMPD3*, *CPPED1*, *KDM5C*, *NIF3L1*, *NUP214*, *RERE*, *SP110*, and *ZNF552*. Five of these genes have been implicated in transcriptional regulation (*TP53*, *KDM5C, RERE, SP110*, and *ZNF552*); whereas two of these genes are known to participate in activation of cell death (*TP53* and *RERE*). The remaining eSNVs were non-synonymous coding variants, predicted to alter the amino acid sequence of the protein product. 

Nine of the 318 novel eSNVs were detected with high confidence in 2 different HER2-positive tumors. We also identified 16 eSNVs that were expressed with high confidence in one or more tumors and with lower confidence (as evidenced by lower depth of coverage at the cognate genomic coordinates) in multiple tumors (Table S9). For example, *NUCB2* is an expressed non-synonymous A to G variant (chromosome 11:17352483). This variant was detected with very high confidence in two tumors (BCT40 with 9 alternate reads and BCT32 with 8 alternate reads) and with reduced confidence in a third tumor (BCT39 with 2 alternate reads).  One non-synonymous eSNV in the *MLL* (*KMT2A*) gene (chromosome 11:118375914) was detected in 4/8 HER2-positive tumors, although at low read depth in 3 of these samples. In addition to these two eSNV, 14 additional candidates corresponding to *TXLNA, HNRNPF, IQGAP2, OTUD7B, GPN3, APS32, WNK4, ANKRD40, RIF1, RPL3, GSK3B, RUVBL1, MRPL3* and *THAP5* genes were detected with high confidence in some samples and at low depth of coverage in other samples (Table S9). 

We interrogated the TCGA exome sequence database to determine if mutations were detected (from exome-seq data) in any of the genes that had stopgain mutations (9 genes) or expressed recurrent eSNVs (16 genes), as described above. As shown in Table S10, we identified 129 TCGA tumors with ERBB2 amplification. Considering the genes with stopgain mutations in our test set, we identified 2 TCGA tumors with KDM5C mutations, 2 with NUP214 mutations, 1 with RERE mutation, 1 with SP110, and 59 with TP53 mutations. Among the genes that expressed recurrent nonsynonymous mutations in our test set, we detected 5 TCGA tumors with MLL (KMT2A) mutations and 2 with RIF1 mutations. ANKRD40, HNRNPF, IQGAP2, OTUD7B, and THAP5 mutations were detected in 1 tumor, each. Somewhat to our surprise, we noticed that many of the tumors that were detected as eSNVs in our test set exhibited copy number variations in TCGA breast tumors. Strikingly ANKRD40 was amplified in 90/129 tumors. This gene is on chromosome 17q21 and not part of the ERBB2 amplicon at 17q12 (Table S10). OTUD7B (1q21.2) was amplified in 92/129 tumors, whereas CPPED1 (16p13.12) and WNK4 (17q21-q22, adjacent to ANKRD40) were amplified in 69/129 and 61/129 tumors, respectively. Thus, eSNV data from our test set of HER2-positive tumors has identified a number of genes that appear to be mutated, both at the single nucleotide sequence and gene copy number levels, in TCGA tumors with ERBB2 amplification. 

### Integrated analysis of HER2-specific genomic features

**Figure 9:**
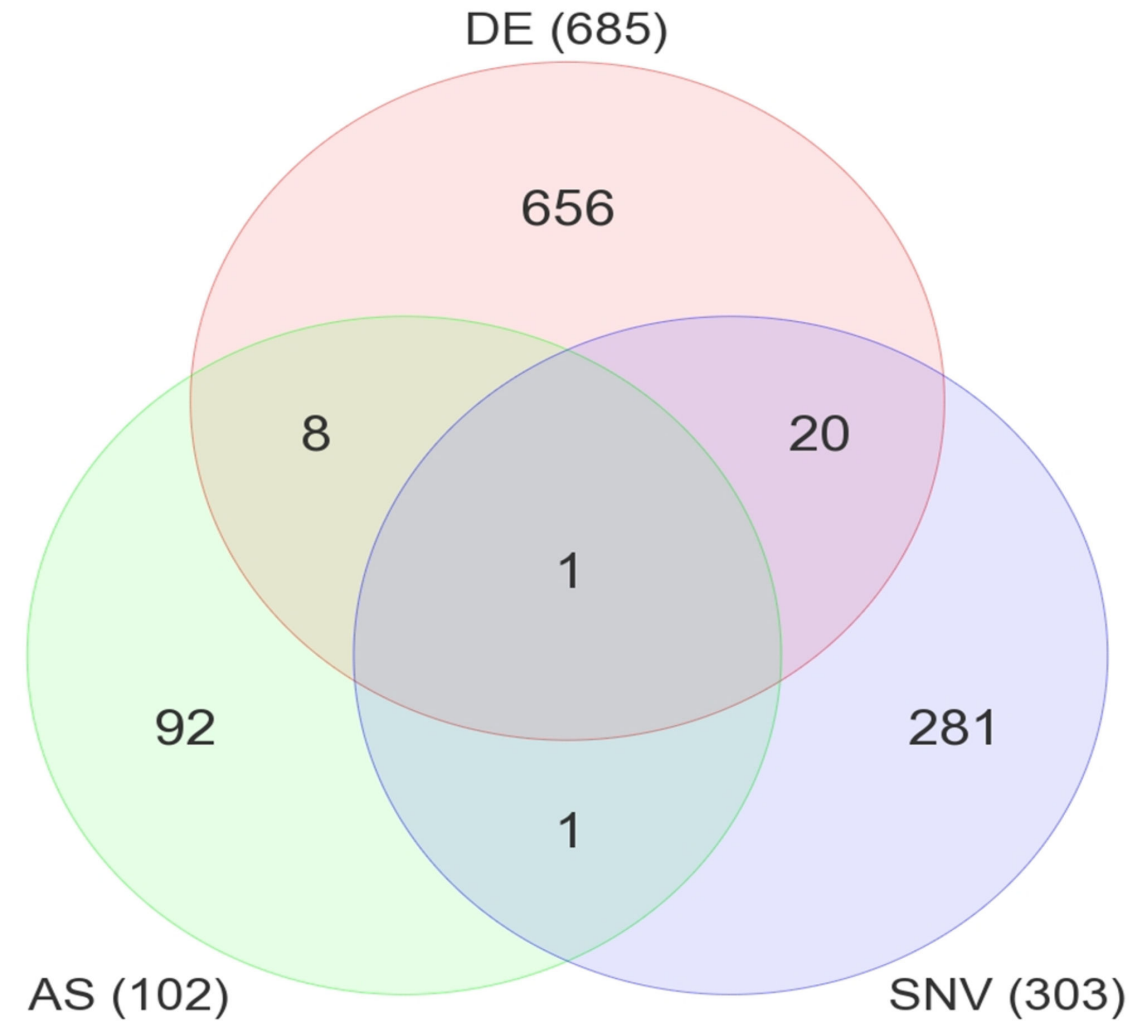
Overlap of genes from genomic features. Venn diagram representation of genes obtained from three genomic features analyses.

**Figure 10:**
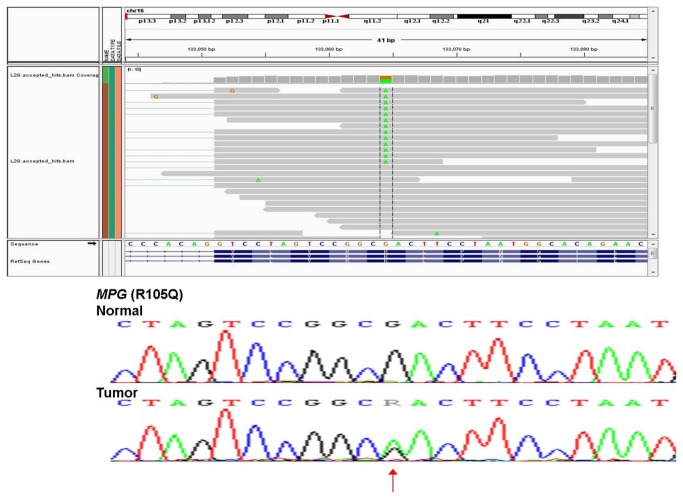
Visualization of single nucleotide variant validation. Sanger sequencing validation of MPG eSNV in HER2 tumor. RNA-Seq reads shown over Sanger sequence tracing with mutation indicated by an arrow.

It should be emphasized that although the genomic features described above are confined to the HER2-positive tumors within our survey panel, we do not wish to imply that these features are HER2-specific in any general sense or that they may have applicability as biomarkers of HER2-positive tumors. Rather, our goal was to define a set of genomic features that were unique to a test set of HER2-positive tumors, to use these features to develop a model of the genomic architecture of the tumors within that test set, and then to test the ability of that model to make predictions about the biological and/or clinical behavior of HER2-positive tumors. Our analyses have identified 685 genes that are differentially expressed in a pattern that is unique to the HER2-positive tumors in our test set of samples. Likewise, we identified 102 genes that are alternatively spliced and 303 genes that contain eSNVs that are uniquely expressed in this panel of HER2-positive tumors. Moreover, our data indicate that there is limited overlap between these genomic features, as illustrated by the VENN diagram in Figure 9. Only 8 of these genes were differentially expressed (DE in Figure 9) and alternatively spliced (AS), whereas 20 of the genes that contain eSNVs (SNV in Figure 9) were also differentially expressed. A single gene, *MPG* (N-methylpurine-DNA glycosylase; EC3.2.2.21), was differentially expressed, alternatively spiced, and contained a non-synonymous somatic mutation. Considering the uniqueness of MPG, we elected to independently validate both the splice variants and the eSNV. RNA-Seq analysis nominated a G to A eSNV (chr16:133064) with 14 reads supporting the alternate allele (A) and 11 reads supporting the reference allele (G). Sanger sequencing confirmed that this is a somatic R105Q (G314A) mutation that appears to be heterozygous in the tumor genomic DNA (Figure 10). 

**Figure 11:**
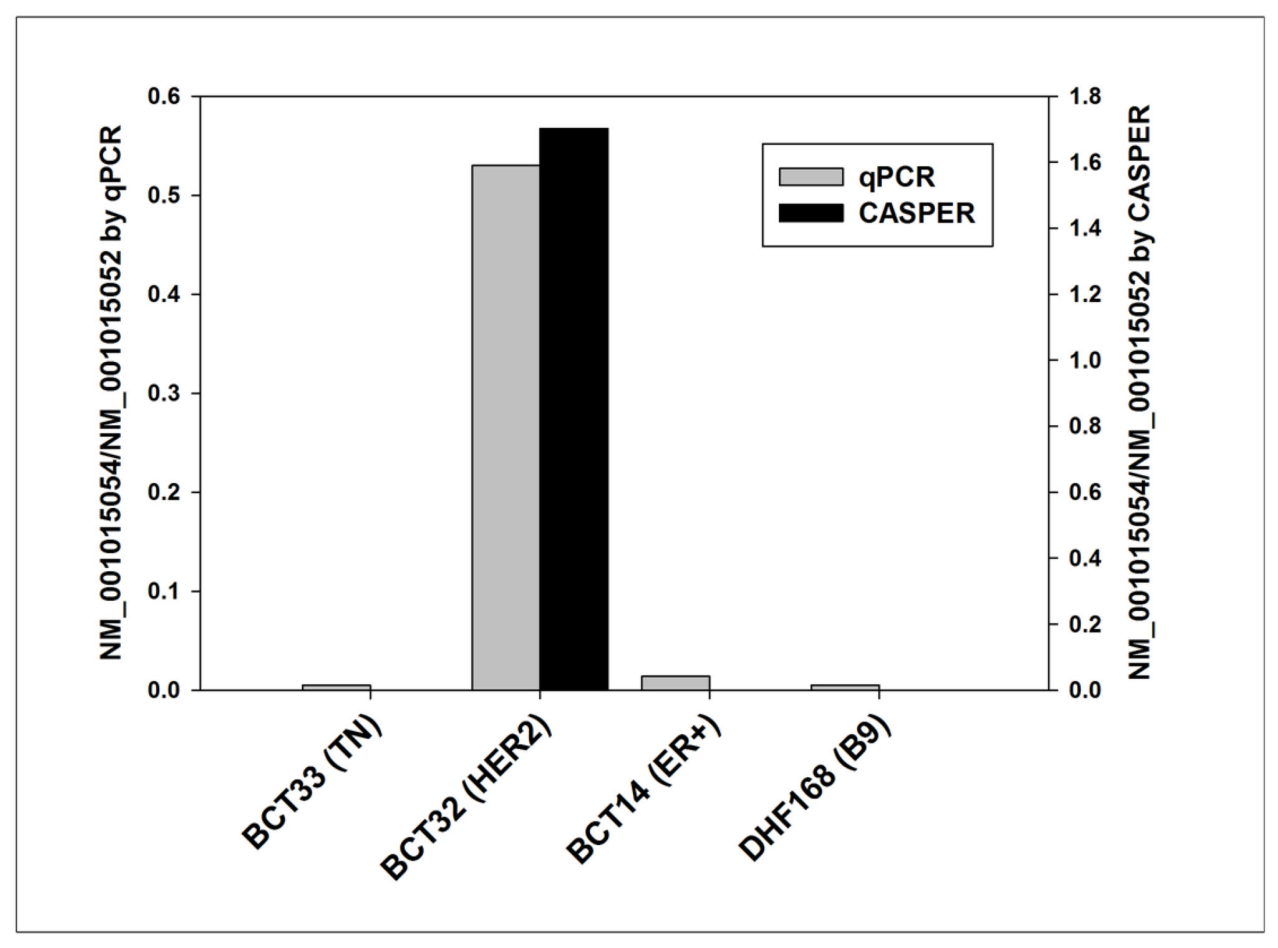
Validation of splicing variants. qPCR validation of the two isoforms in breast tumor samples for MPG splicing variants. Isoform abundance by qPCR is indicated by gray bars, whereas isoform abundance determined by CASPER is shown in black bars.

RNA-Seq analysis indicated that 2 isoforms of the MPG (NM_001015052 and NM_001015054) were expressed in breast tumors. CASPER predicted that the NM_001015054 splice form was expressed at high levels uniquely in our HER2-positive tumors, compared to NM_001015052, as illustrated by the black bars in Figure 11. Overexpression of NM_0010154 in the HER2-positive test set was confirmed using isoform-specific qPCR primer/probes, as shown by the gray bars in Figure 11.

**Figure 12:**
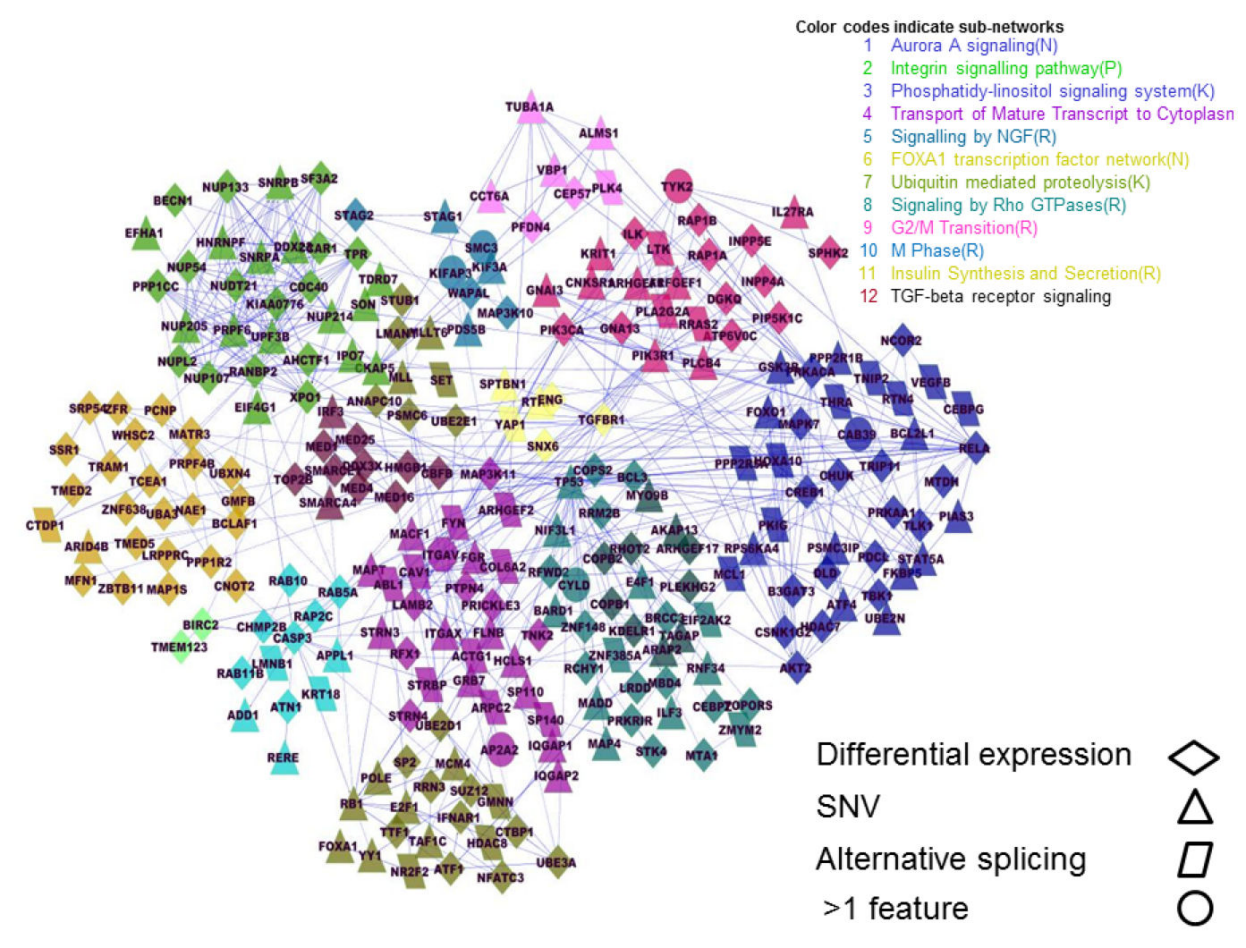
HER2-positive tumor interactome. HER2 tumor interactome network developed using cytoscape and functional reactome of 1055 genes obtained from integration analyses of genomic features. Different functional modules within the network are color coded.

 The 1090 genomic features that we have identified in our HER2 tumor panel (685 differentially expressed, 102 alternatively spliced, 303 eSNVs) correspond to 1055 genes. We next asked whether any or all of these genes interact in a manner that might define processes that are associated with the HER2-positive tumors in our sample cohort. To this end we used the Reactome FI feature of Cytoscape to build an interactome map that incorporates genes that encode proteins with known functional interactions [29]. Reactome FI generated a highly integrated network of 244 genes with 541 edges (connections), as shown in Figure 12. To test for random association, we ran 20 Cytoscape simulations using 1055 genes selected at random from the dataset. The mean number of genes integrated into these random networks was 108 (standard deviation = 22) and the mean number of connections generated at random was 175 (standard deviation = 39). Thus the level of integration that we observed within the network shown in Figure 12 (244 genes with 541 edges) was >5 standard deviations above the mean predicted for random association. Bearing in mind that these edges are defined by known interactions, and therefore likely to have functional significance, it is notable that 80 of the nodes within the overall network have 5 or more connections; whereas 32 nodes have 10 or more connections (Table S11). 

The interactome model shown in Figure 12 is comprised of 12 functionally discrete sub-networks or modules. A high degree of connectivity is apparent within the individual modules, each of which is, in turn, linked to the integrated network through multiple module to module connections. Functional annotation of the modules is given in Table 1, and can be broadly summarized as signal transduction and transcription (modules 2, 3, 5, 6, 8, and 12); protein synthesis, degradation, and secretion (modules 7 and 11); RNA processing (module 4); and processes associated with G2/M phase of the cell cycle (modules 1,9, and 10). Given that this network accommodates a set of genomic features that are associated with the HER2-positive tumors in our samples, we posit that these processes represent a set of interconnected functions that are critical to the establishment and/or maintenance of the HER2-positive tumor phenotype.

**Table 1:** Sub networks from the HER2-positive tumor interactome model.

**Module**	**Pathway**	**Number of Genes in Module**	**Number of Genes in Pathway**	**P-value**	**FDR Pathway Enrichment**	**Nodal Genes in Pathway**
1	Aurora A signaling (N)	44	6	0	<1.00e-03	CYLD, BCL3, CHUK, RELA, TP53, BIRC2
2	Integrin signaling pathway (P)	33	10	0	<1.00e-03	CAV1, ACTG1, LAMB2, ITGAX, ARPC2, ITGAV, COL6A2 ,FLNB, FYN, ABL1
3	Phosphatidyl-inositol signaling system(K)	33	7	0	<1.00e-03	PIP5K1C, PLCB4, PIK3CA, INPP5E, PIK3R1, DGKQ, INPP4A
4	Transport of Mature Transcript to Cytoplasm (R)	27	10	0	<1.00e-03	NUP214, CDC40, NUP54, RANBP2, TPR, NUP133, NUPL2, UPF3B, NUP205, NUP107
5	Signaling by NGF(R)	24	8	0	<1.00e-03	PPP2R1B, RTN4, CREB1, FOXO1, ATF1, GSK3B, PRKACA, AKT2
6	FOXA1 transcription factor network (N)	23	2	0.0009	2.26E-01	FOXA1, NR2F2
7	Ubiquitin mediated proteolysis (K)	17	5	0	<1.00e-03	UBE3A, ANAPC10, STUB1, UBE2D1, UBE2E1
8	Signaling by Rho GTPases (R)	9	6	0	<1.00e-03	TAGAP, PLEKHG2, RHOT2, ARHGEF17, AKAP13, ARAP2
9	G2/M Transition (R)	9	5	0	<1.00e-03	PLK4, CEP57, CKAP5, ALMS1, TUBA1A
10	M Phase (R)	8	3	0	<1.00e-03	SMC3, STAG2, STAG1
11	Insulin Synthesis and Secretion (R)	7	3	0	1.00E-03	SRP54, TRAM1, SSR1
12	TGF-beta receptor signaling	6	4	0	<1.00e-03	SNX6, TGFBR1, YAP1, ENG

The interactome model identified 12 discrete modules or sub-networks. The total number of genes in each module as well as key nodal genes within each module are given below.

### Confirmation of pathway enrichment in TCGA samples

The genomic landscape model described above was generated from a survey panel containing 8 HER2-positive tumors. It is therefore relevant to ask to what extent the pathways described above can be applied generally to HER2-positive tumors. To address this issue, we used gene expression data (RNA-Seq gene counts) from TCGA breast tumors to determine if any or all of the 12 pathways described above was enriched in HER2-positive tumors from a much larger dataset. Initially, we summarized p-values for differential expression, in the TCGA samples, of genes that had been identified as differentially expressed in our study cohort. As shown in Figure S2, we observed that these genes were significantly enriched, based on distribution of p-values, in comparisons of HER2 to tissue adjacent normal and to basal tumors. A significant trend towards enrichment was observed in the comparison of HER2 versus luminal tumors. We then used Fisher’s exact test to determine the extent to which genes that were incorporated in the 12 Cytoscape network pathways were enriched in TCGA samples, relative to genes that were identified as differential features in HER2-positive tumors, but were not incorporated into any of the Cytoscape pathways. As shown in Table 2, genes associated with all 12 pathways were significantly enriched in the comparison of HER2-positive versus normal; whereas 11 out of 12 pathways were enriched when HER2-positive samples were compared to basal tumors. Integrin signaling, ubiquitin-mediated proteolysis, and TGFbeta receptor signaling were significantly enriched in the comparison of HER2-positive versus luminal tumors. We suspect that the relatively lower level of enrichment in the comparison with luminal tumors (in contrast to HER2 vs basal) reflects the well-known overlap between the clinical HER2-positive definition, which we used in our initial analysis, and the luminal intrinsic subtype used for TCGA data. Nevertheless, genes associated with integrin signaling, ubiquitin-mediated proteolysis, and TGF-beta receptor signaling were significantly enriched in HER2-positive versus normal, basal, and luminal samples from TCGA, thereby providing supporting evidence for our conclusion that integrin signaling has some critical role in HER2-positive tumors, compared to the other forms of breast cancer. 

**Table 2:** Pathway enrichment statistics in breast cancer subtypes from TCGA.

			**Differential Expression at p<0.05 in pairwise comparsions**					
**PathNo.**	**Pathway Description**	**Genes in Pathway**	**HER2 vs Normal Genes**	**p-value**	**HER2 vs Basal Genes**	**p-value**	**HER2 vs Luminal Genes**	**p-value**
p01	Aurora A signaling(N)	44	27	2.99E-08	25	8.46E-07	15	9.83E-02
p02	Integrin signalling pathway(P)	33	23	9.59E-09	20	2.93E-06	16	1.19E-03
p03	Phosphatidy-linositol signaling system(K)	33	23	9.59E-09	19	1.53E-05	11	2.05E-01
p04	Transport of Mature Transcript to Cytoplasm(R)	27	18	1.27E-06	17	8.32E-06	9	2.45E-01
p05	Signalling by NGF(R)	24	20	4.36E-10	13	8.41E-04	8	2.27E-01
p06	FOXA1 transcription factor network(N)	23	14	9.59E-05	10	4.10E-02	2	1.32E-01
p07	Ubiquitin mediated proteolysis(K)	17	14	3.18E-07	11	2.67E-04	8	3.59E-02
p08	Signaling by Rho GTPases(R)	9	8	5.39E-05	6	6.43E-03	1	6.93E-01
p09	G2/M Transition(R)	9	6	6.43E-03	7	7.67E-04	4	2.24E-01
p10	M Phase(R)	8	5	1.92E-02	6	2.65E-03	3	3.96E-01
p11	Insulin Synthesis and Secretion(R)	7	5	8.85E-03	4	5.40E-02	2	6.65E-01
p12	TGF-beta receptor signaling	6	4	2.82E-02	4	2.82E-02	4	2.82E-02

Fisher’s Exact test was carried out using a 2X2 contingency table to compare the number of genes from the Ctyoscape network that were differentially expressed in TCGA tumors compared to the remaining genes that were defined as “HER2-specific” in our analysis but were not incorporated into the Cytoscape network. The contingency table was constructed as follows: The constants were the total number of genes (G, 1055 genes from our initial analysis), the total number of genes from this set that were incorporated into the cytoscape network (N, 244 genes), and the total number of genes in the specific pathway to be analyzed (P). For each pathway, we compared the number of genes within that pathway that were differentially expressed at p<0.05 in the TCGA data (m) to the remainder of genes in the candidate list (N-m) versus the genes in the pathway that were not differentially expressed (P-m) against the number of genes that were in neither the candidate list nor the pathway (G-N-P+m).

### Potential clinical significance of the HER2 interactome map

#### Linking the model to response to HER2-targeted therapy in vitro

Landscape models of the sort shown in Figure 12 can be used to define processes that are critical to the pathology of tumors. However, from a translational standpoint, such models are useful to the extent that they can be linked to therapeutic response. Initially, we asked if any of the genes from our network modules might be associated with response to abrogation of HER2 signaling. To this end we used our landscape model genes to interrogate data from the recently published Cell Line Encyclopedia [31], which includes both gene expression (Affymetrix arrays) and sensitivity (EC50) to the HER2 small molecule tyrosine kinase inhibitor lapatinib in 18 established breast cancer cell lines. Rank order correlation was used to determine if expression of any of our 244 HER2-associated genes from the interactome map correlated with response to HER2-targeted therapy in vitro. It should be noted that in this analysis, and those described below, we are in some cases interrogating differences in mRNA abundance of genes that were initially identified based on eSNV or alternative splicing. This approach was adopted based on the rationale that a gene that might be activated or suppressed by mutation or alternative splicing in a given tumor might also be activated or repressed by altered expression in a different tumor. In all of these analyses we are asking if the pathway is important, irrespective of the precise mechanism ascribed to individual genes within the pathway. The validity of this proposition is re-enforced by the observation that many of the genes that we detected as eSNVs in our survey panel were overexpressed due to gene amplification in TCGA breast samples. Using this approach, we identified 26 genes whose expression correlated with lapatinib response (Table 3). Monte Carlo simulation suggest that the probability that such a correlation might occur at random is <0.001. The most significant of these was integrin-linked kinase (ILK, p<0.0001). Genes involved in RHO-family GTPase signaling and M-phase progression were also prominent in this cohort. Broadly speaking, these data suggest that response to HER2-targeted therapy is likely to be influenced not only by ERBB2 (the target of such therapy) but also by the pathways that are associated with HER2-mediated transformation. Among these pathways, our data indicate that integrin signaling, processes associated with M-phase progression, and RHO-family GTPase signaling are associated with, and perhaps likely to impinge upon, the extent to which cells respond to abrogation of HER2 signaling. 

**Table 3:** Correlation of genes with lapatinib response in breast cancer cell lines.

**ID**	**Gene Description**	**Type**	**r(Spearman)**	**p-value**	**Function**
ILK	integrin-linked kinase	DE	-0.791538	9.10E-05	Integrin signaling
ALMS1	Alstrom syndrome 1	SNV	0.76677	2.00E-04	cell transport, microtubules
CDC40	cell division cycle 40 homolog (*S. cerevisiae*)	DE	0.680083	1.90E-03	RNA processing
STAG2	stromal antigen 2	DE	0.673891	2.17E-03	M-phase
MBD4	methyl-CpG binding domain protein 4	DE	0.636739	4.49E-03	Mismatch repair
ANAPC10	anaphase promoting complex subunit 10	DE	0.622291	5.82E-03	M-phase
ATF1	activating transcription factor 1	DE	0.587203	1.04E-02	cAMP signaling
ZNF385A	zinc finger protein 385A	AS	-0.581011	1.15E-02	unknown (transcription?)
NUP54	nucleoporin 54kDa	DE	0.560372	1.56E-02	Nuclear pore comples
APPL1	adaptor protein, phosphotyrosine interaction, PH domain and leucine zipper containing 1	SNV	-0.547988	1.86E-02	RHO-GTPase signaling
NFATC3	nuclear factor of activated T-cells, cytoplasmic, calcineurin-dependent 3	DE	0.54386	1.96E-02	Calcium signaling
MCL1	myeloid cell leukemia sequence 1 (BCL2-related)	AS	0.54386	1.96E-02	Apoptotic regulation
RAB10	RAB10, member RAS oncogene family	DE	0.539732	2.08E-02	RHO-GTPase signaling
VBP1	von Hippel-Lindau binding protein 1	SNV	0.527348	2.45E-02	Protein folding
ARFGEF1	ADP-ribosylation factor guanine nucleotide-exchange factor 1(brefeldin A-inhibited)	SNV	-0.517028	2.80E-02	RHO-GTPase signaling
RFX1	regulatory factor X, 1 (influences HLA class II expression)	DE	0.517028	2.80E-02	Transcriptional regulation
RAP1A	RAP1A, member of RAS oncogene family	DE	0.508772	3.11E-02	RHO-GTPase signaling
RRN3	RRN3 RNA polymerase I transcription factor homolog (*S. cerevisiae*)	DE	0.506708	3.19E-02	Transcriptional regulation
UPF3B	UPF3 regulator of nonsense transcripts homolog B	SNV	0.504644	3.27E-02	RNA processing
UBE2E1	ubiquitin-conjugating enzyme E2E 1	DE	0.498452	3.53E-02	Protein processing
TPR	translocated promoter region	DE	-0.494324	3.70E-02	M-phase
LAMB2	laminin, beta 2 (laminin S)	DE	-0.49226	3.80E-02	Extracellular matrix/adhesion
NIF3L1	NIF3 NGG1 interacting factor 3-like 1 (*S. pombe*)	SNV	0.490196	3.89E-02	unknown (transcription?)
PRKACA	protein kinase, cAMP-dependent, catalytic, alpha	DE	0.479876	4.39E-02	cAMP signaling
PPP1R2	protein phosphatase 1, regulatory subunit 2	DE	0.477812	4.49E-02	Protein phosphorylation
ZNF638	zinc finger protein 638	DE	0.475748	4.60E-02	Transcriptional regulation

Correlation of gene expression with lapatinib response in breast cancer cell lines from a freely available data source (Cancer Cell Line Encyclopedia project) using the list of genes obtained from our HER2 interactome network.

#### Linking the model to taxane sensitivity in-vitro

Several large clinical trials have demonstrated that in the adjuvant setting, the efficacy of HER2-targeted therapy is enhanced by concurrent treatment with taxanes [34]. We therefore asked whether our HER2 transcriptome model might identify potential mechanistic links between HER2 signaling and response to therapies that target processes that are involved in maintenance of the cytoskeletal architecture (e.g., tubulin, the target of taxanes). Thus we used our landscape model genes to interrogate gene expression (Affymetrix arrays) and paclitaxel sensitivity (EC50) for breast cancer cell lines from the Cell Line Encyclopedia dataset. Rank order correlation identified 17 genes that correlated with p<0.05, as shown in Table 4. Four of these genes (RAB11B, IQGAP1, TAGAP, and PLEKHG2) are involved in RHO-family GTPase signaling and therefore potentially involved in remodeling of the cytoskeleton. Four other genes are also involved in regulation of the cytoskeleton: CCT6A is a chaperon involved in tubulin folding, whereas SON is involved in tubulin splicing; KDELR1 is involved in vesicular transport, a process that is linked to the tubulin cytoskeleton; and KIFIP3 is involved in cytokinesis, which likewise depends on cytoskeletal architecture. We found that eight-seventeenths of the HER2-associated genes that correlate with paclitaxel sensitivity are also involved in regulating the dynamics of the cytoskeleton. The data suggest that a subset of HER2-associated genomic features may contribute to the well-known therapeutic efficacy of combining therapies that simultaneously target growth factor signaling (trastuzumab) and cytoskeletal processes (paclitaxel). We used Monte Carlo simulation to evaluate the possibility that the associations which we described might arise at random. Initially, we noted that six-seventeenths of the genes that correlated with paclitaxel sensitivity were identified (by GO terms) as being involved in signal transduction (GO:0007165). We ran 1000 simulations using 244 genes selected at random from the dataset and determined that the likelihood that any six genes would correlate with paclitaxel sensitivity and also map to a single broad biological process was <0.001. Based on this observation, we conclude that the observation that 8/17 genes have known functions related to cytoskeletal dynamics is very unlikely to be due to random association. 

**Table 4:** Correlation of genes with paclitaxel response in breast cancer cell lines.

**ID**	**Gene Description**	**Degree**	**Type**	**r(Spe-arman)**	**p-value**	**Function**
CCT6A	chaperonin containing subunit 6A	3	SNV	-0.60	5.31E-03	tubulin folding
ATN1	atrophin 1	2	DE	0.59	6.07E-03	transcriptional co-regulator
STRN4	striatin, calmodulin binding protein 4	2	DE	0.54	1.34E-02	caclium signaling
RAB11B	RAB11B, RAS oncogene family	2	DE	0.52	1.79E-02	RHO-GTPase signaling and endosomal trafficking
NR2F2	nuclear receptor subfamily 2, group F, member 2	4	AS	0.51	2.12E-02	nuclear receptor activated by retinoids
TOPORS	topoisomerase I binding, arginine/serine-rich	1	DE	0.51	2.26E-02	ubiquitin ligase regulates TP53 turnover
IQGAP1	IQ motif containing GTPase activating protein 1	2	SNV	0.51	2.31E-02	RHO-GAP associated with cytoskeletal reorganization
TGFBR1	transforming growth factor,betareceptor1	10	DE	-0.50	2.61E-02	TGF-beta signal transduction
CASP3	caspase 3, apoptosis-related cysteine peptidase	10	DE	-0.50	2.61E-02	apoptotic signaling
KIFAP3	kinesin-associated protein 3	2	DE,AS	0.49	2.88E-02	cytokinesis
SF3A2	splicing factor 3a, subunit 2, 66kDa	9	DE	0.49	2.99E-02	RNA splicing
MAP3K10	mitogen-activated protein kinase kinase kinase 10	2	DE	0.47	3.49E-02	activates JNK signaling
TAGAP	T-cell activation RhoGTPase activating protein	1	SNV	0.46	4.20E-02	RHO-GTPase signaling
SON	SON DNA binding protein	1	SNV	-0.45	4.43E-02	RNA splicing, tubulin
KDELR1	KDEL endoplasmic reticulum protein retention receptor 1	2	DE	0.45	4.43E-02	vesicular transport
SMARCE1	SWI/SNF related, matrix associated, actin dependent regulator of chromatin, subfamily e, member 1	6	DE	-0.45	4.51E-02	chromatin remodeling, ER signaling
PLEKHG2	pleckstrin homology domain containing, family G member 2	1	DE	0.45	4.84E-02	RHO-GTPase signaling

Correlation of gene expression with paclitaxel response in breast cancer cell lines from Cancer Cell line Encyclopedia project using HER2 interactome genes.

#### Linking the model to adjuvant trastuzumab therapy in patients

The ultimate goal of integrated genomic analysis of this sort is to create a model that has predictive value for clinical management of cancer patients. We took advantage of a recently-developed set of genomic data derived from the NCCTG N9831 clinical trial, in which DASL microarray technology was used to quantify mRNA abundance in 488 patients with HER2-positive tumors treated concurrently with trastuzumab (Herceptin®) plus paclitaxel in the adjuvant setting [12]. A manuscript describing the analysis of the N9831 data (Perez et al. “Genomic analysis reveals that immune function genes are strongly linked to clinical outcome in the NCCTG (Alliance) N9831 adjuvant trastuzumab trial”) is currently under review. Geneset Analysis [32] was used to determine if any of the modules described in the HER2 interactome map correlated with risk of recurrence (using time to event as a continuous variable). As shown in Table 5, module 2 (integrin signaling) was highly associated with risk of relapse (p=0.003, FDR=0.04). There was a tendency for module 8 (RHO-GTPase signaling, p=0.07) and module 12 (TGF-beta signaling, p=0.06) to correlate with relapse. 

**Table 5:** Gene set analysis of 12 sub-network genes with N9831 clinical trial data.

			**Arm C**		
Gene_set_name	Pathway	Score	p-value	FDR	pos.neg.vector
HER2.network.01	Aurora A signaling(N)	-0.1	0.1756	0.571	negative
HER2.network.02	Integrin signalling pathway(P)	-0.555	0.0032	0.042	negative
HER2.network.03	Phosphatidy-linositol signaling system(K)	0.0859	0.2798	0.548	positive
HER2.network.04	Transport of Mature Transcript to Cytoplasm(R)	0.0316	0.4296	0.621	positive
HER2.network.05	Signalling by NGF(R)	0.2084	0.1126	0.366	positive
HER2.network.06	FOXA1 transcription factor network(N)	0.0936	0.2948	0.548	positive
HER2.network.07	Ubiquitin mediated proteolysis(K)	0.3351	0.0344	0.366	positive
HER2.network.08	Signaling by Rho GTPases(R)	-0.486	0.0739	0.48	negative
HER2.network.09	G2/M Transition(R)	0.1197	0.3491	0.567	positive
HER2.network.10	M Phase(R)	-0.266	0.1583	0.571	negative
HER2.network.11	Insulin Synthesis and Secretion(R)	0.2276	0.2188	0.548	positive
HER2.network.12	TGF-beta receptor signaling	0.512	0.0611	0.366	positive
HER2.network.all			0.0936	0.366	positive

The direction of the GSA score indicates the relationship to outcome, as does the column entitled “pos.neg.vector”. Negative indicates an association with increased risk of relapse, whereas positive indicates decreased risk of relapse. GSA was carried out using gene expression data and outcomes (relapse free survival) as continuous variables.

## Discussion

The studies described in this report were motivated by two central hypotheses. The first of these was that we could integrate multiple genomic features from RNA-Seq data to model the genomic architecture of a test set of HER2-positive tumors. The second hypothesis was that this model would make relevant biological predictions about HER2-positive tumors from other sample cohorts. We have identified over 1000 genes that potentially manifest differential activity in the test set of HER2-positive tumors, either at the level of expression (mRNA abundance) or nucleotide sequence (alternative splicing or eSNV). These genomic features are organized into distinct functional processes that appear to be critical to establishment and maintenance of the HER2-positive phenotype in our test set of tumors. The generality of our model is substantiated by the observation that many of the functional processes defined by this model are also enriched in HER2-postive tumors from TCGA. The predictive potential of the model is evidenced by the link between the functional process identified in the test set and both cellular and clinical properties of HER2-positive cells and tumors. This report represents, to our knowledge, the first attempt to use RNA-Seq data to develop a functional interactome map that integrates multiple genomic features in HER2-positive breast tumors. Having said that, it must be emphasized that this is a “first draft” model, built using a survey panel of tumors. Development of a more comprehensive model will require analysis of a much larger panel of tumors, and this effort is currently ongoing. 

 From the standpoint of tumor biology, the interactome model that we have developed makes several novel and testable predictions about the processes that underlie the HER2 phenotype. Most obvious of these is the potential role of integrin signaling, which has not been previously evaluated in depth in cells derived from HER2-positive tumors. The implications of RHO-family GTPase signaling and processes linked to the cytoskeleton (e.g. M-phase progression) are equally provocative. A key question, which cannot be easily answered from a systems biology model of the sort that we have developed, is whether these processes are activated or repressed in HER2-positive tumor cells. The network predicts that these processes are important, but directionality must be assessed in vitro. 

 We used two independent approaches to test the hypothesis that these HER2-associated genomic features and processes are informative of the manner in which HER2-positive tumors or breast cancer cells respond to therapy. Initially, we utilized the Cell Line Encyclopedia dataset, which contains gene expression and drug sensitivity data for a panel of established breast cancer cells. We initially looked for genes from our interactome map that correlated with lapatinib sensitivity in vitro. We appreciate that there are significant limitations to this approach including the facts that lapatinib is not uniquely a HER2 inhibitor, the cell lines that we interrogated were not primarily derived from HER2-positive tumors, and the data that were available for those cells was limited to mRNA abundance. However, lacking a comprehensive set of data on trastuzumab sensitivity in vitro, we asked if any of the genomic features that were identified using HER2-positive tumors correlated with inhibition of cell proliferation by lapatinib in vitro. We identified 26 genes including integrin linked kinase (ILK). A number of genes linked to RHO-family GTPase and M-phase progression were also identified, consistent with our hypothesis that HER2-positive tumors manifest processes that impinge on organization and function of the subcellular architecture.

 The standard of care for HER2-positive tumors involves combined HER2-targeted and microtubule-targeted therapy with taxanes. We therefore interrogated the Cell Line Encyclopedia data to test the hypothesis that HER2-positive tumors manifest unique features that are associated with sensitivity to taxanes (paclitaxel). We identified 17 HER2-associated genes that correlate with taxane sensitivity, including a number of genes that are linked to functions related to the subcellular architecture (RHO-family GTPase signaling and microtubule dynamics). 

 Overall, the data from cell lines are consistent with our hypothesis that the HER2-positive tumor interactome map identifies features and processes that predict the biological properties of HER2-positive tumor cells, as assessed by response to lapatinib and paclitaxel in vitro. However, the critical test of the hypothesis is inherent in the extent to which the processes that we have identified are informative of therapeutic response in the clinic. To test this hypothesis, we used gene set analysis to determine if any of the 12 modules from the interactome map was associated with risk of relapse in the N9831 clinical trials specimens. Integrin signaling was associated with risk of relapse with a very high degree of statistical significance. RHO-family GTPase signaling and TGF-beta signaling exhibited a trend towards association with relapse-free survival in the N9831 samples. Extension of our findings to a larger dataset (TCGA) emphasizes the potential significance of integrin signaling, ubiquitin-mediated proteolysis, and TGF-beta receptor signaling in HER2-positive breast cancer, and suggests that processes linked to M-phase may also be of particular importance in this breast cancer subtype. 

 In conclusion, we have shown that there are functional genomic process that are consistently associated with HER2-positive tumors, that these processes can be elucidated by an integrated analysis of multiple genomic features, and that some of these processes appear to predict both biological and clinical properties of HER2 tumor cells and HER2-positive tumors. The most striking evidence of this assertion is, perhaps, the identification of a strong and previously unappreciated link between integrin signaling and response to HER2-targeted therapy. Our data suggest that surrogate markers of integrin signaling (e.g. ILK) may be useful as predictors of therapeutic efficacy. Two significant challenges inform our future directions. The most obvious of these challenges is to refine the HER2-positive tumor transcriptome landscape model using a larger cohort of samples for which mRNA abundance, alternative splicing, and eSNVs can be interrogated. The second challenge is to dissect the functionally significant modules within this model and identify new therapeutic targets that can be developed for treatment of patients who have failed HER2-targeted therapy. Achievement of this aim will obviously require analysis of pre-clinical models, and studies to this end are currently underway.

## Supporting Information

Figure S1 **Normalization plots.** Before and after normalization plots of gene expression counts for samples.
(TIF)

Figure S2 **Enrichment of differentially expressed genes in TCGA.** DTK was used to calculate p-values for differential expression, within TCGA samples, of genes that had been identified as differentially expressed in HER2+ tumors from the initial analysis. The frequency distribution of p-values for all genes in each of the subtypes is shown.
(TIFF)

Table S1 **Sample characteristics.** Clinical/pathological characteristics of these tumors sequenced.
(XLSX)

Table S2 **Alignment statistics.** Summary of alignment statistics for 8 ER+, 8 TN, 8 HER2 and 8 benign breast lesion samples.
(XLSX)

Table S3 **Differentially expressed genes.** List of 685 genes that are differentially expressed in HER2-positive tumor samples compared to other groups with a p-value <0.05.
(XLSX)

Table S4 **List of splicing variants.** List of 199 transcripts that are alternately spliced in HER2-positive tumors compared to other groups.
(XLSX)

Table S5 **The eSNVs in HER2-positive tumors.** List of 318 expressed SNVs (eSNvs) that are expressed in HER2 tumors compared to other tumor subtypes.
(XLSX)

Table S6 **Genes associated with eSNVs in HER2-positive tumors.** List of 303 genes corresponding to 318 eSNVs in HER2-positive tumors.
(XLSX)

Table S7 **Summary of eSNVs.** Summary of eSNVs per sample and per breast tumor subtype.
(XLSX)

Table S8 **Summary of Sanger sequencing validation results.** Sanger sequencing details of 83 high confident eSNVs nominated in a HER2-positive tumor sample (BTC40) for validation.
(XLSX)

Table S9 **List of eSNvs in HER2-positive tumors.** List of low and high confidence eSNVs that were detected in HER2-positive tumors.
(XLSX)

Table S10 **Investigation of eSNVs genes with TCGA exome sequencing and copy number data.** Summary of somatic SNVs and CNVs from TCGA exome sequening data in 25 genes with stop gain or nonsynonymous SNVs from our survey panel.
(XLSX)

Table S11 **Transcriptome landscape statistics.** Network statistics of nodes or genes with at least 5 more connections in the HER2 integrated network.
(XLSX)
