# Supplementary figures and images for: An Integrated Model of the Transcriptome of HER2-Positive Breast Cancer
Source: PLoS One. 2013 Nov 1;8(11):e79298. doi: 10.1371/journal.pone.0079298 (PMC3815156; doi:10.1371/journal.pone.0079298)

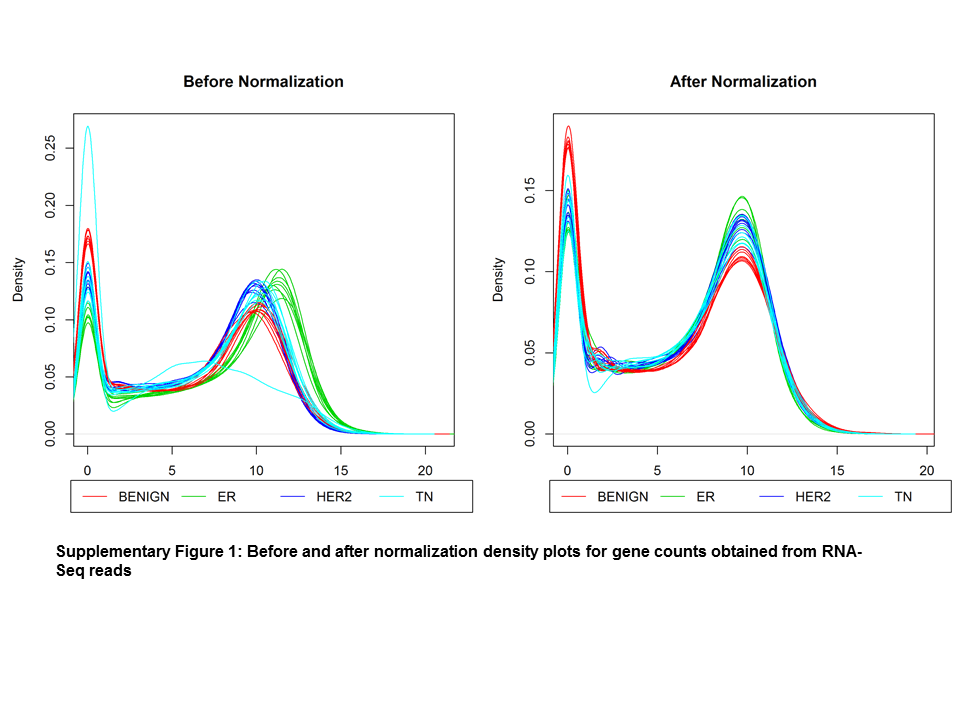

Supplement: Figure S1 — Normalization plots. Before and after normalization plots of gene expression counts for samples. (TIF) [file pone.0079298.s001.tif]

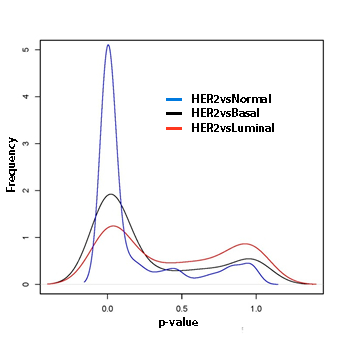

Supplement: Figure S2 — Enrichment of differentially expressed genes in TCGA. DTK was used to calculate p-values for differential expression, within TCGA samples, of genes that had been identified as differentially expressed in HER2+ tumors from the initial analysis. The frequency distribution of p-values for all genes in each of the subtypes is shown. (TIFF) [file pone.0079298.s002.tiff]
